# Evaluating the quality of online fertility nutrition claims

**DOI:** 10.1017/S1368980025100876

**Published:** 2025-08-12

**Authors:** Kimberly R. Lush, Amy T. Hutchison, Jessica A. Grieger

**Affiliations:** 1 Adelaide Medical School, Faculty of Health and Medical Sciences, The University of Adelaide, Adelaide, SA, Australia; 2 Robinson Research Institute, The University of Adelaide, Adelaide, SA, Australia

**Keywords:** Nutrition claims, Information quality, Fertility nutrition, Health communication, Content analysis

## Abstract

**Objective:**

To (1) explore and analyse current online preconception health and nutrition-related claims, (2) assess identified online preconception health claims against current preconception guidelines and (3) understand the perceived health claims among reproductive-aged men and women.

**Setting:**

Five online media platforms were searched using fertility nutrition-related search terms.

**Participants:**

All claims were assessed by an expert panel against nine Australian and International preconception guidelines. A sample of eighty reproductive-aged men and women rated a random sample of claims.

**Design:**

A content analysis of 191 claims was conducted using NVivo 12 Plus to group recurring topics into themes and then categories. Survey participants rated forty claims using a 5-point Likert scale from ‘Not at all likely’ to ‘Highly likely’. If at least 75 % of the surveyed population considered a claim ‘likely’ or ‘unlikely’, it was classified as such.

**Results:**

Two themes were generated: *nutrition claims* and *lifestyle claims*. Five percent of claims were present in preconception guidelines, while 54 % had no evidence to support the claim. The highest percentage of no evidence claims was for *whole foods and their components* and *dietary patterns*. TikTok and Instagram contained the highest proportion of non-evidence-based claims. The community considered 3/40 claims likely to be true and 3/40 claims unlikely to be true.

**Conclusions:**

There is a myriad of inaccurate information online related to fertility nutrition and lifestyle behaviours. Social media public health campaigns to disseminate quality evidence for preconception health are necessary to improve awareness among those who access online information.

Social media and online resources are increasingly used as a method of health information transmission^(1–3)^, with multiple pages and websites dedicated to specific health conditions. Dietitians, nutritionists and general practitioners are often listed as the three most preferred sources of nutrition information and are perceived to be the most trustworthy, credible and effective^(4)^. Specifically for women planning a pregnancy, the ease of access to the internet allows them to search for health-related information both before and after, consulting with health professionals^(5)^. It has been shown that women in the preconception period or in early pregnancy utilise the internet at least once per month to find health-related information; however, women report not discussing this information with their healthcare providers as they perceive the information online to be reliable and useful^(6)^. This has the potential to create an environment where inaccurate information or mistaken beliefs may not be corrected.

A systematic review of online, nutrition-related information found that most content was inaccurate and of low quality. According to a panel of experts, only 17 % of the forty-seven assessed websites and social media posts contained accurate information^(7)^. Similarly, an analysis of 676 nutrition-related Instagram posts found that only 6 % were of ‘good’ quality, with none rated as ‘excellent’ when using the Principles for Health-Related Information on Social Media tool^(8)^. No study has specifically reviewed online nutrition-related claims in regards to preparing for conception. Concerningly, recent research indicates that a majority of fertility-related social media posts are not authored by health professionals^(9)^, and much of the conception-related health information found online is considered inaccurate by fertility and conception experts^(10)^. For example, in a 2019 study, Kedzior *et al.* ^(10)^ reviewed and categorised eighty-nine claims into three themes: ‘conception behaviour and monitoring’, ‘lifestyle and exposures’ and ‘medical’. While 40 % of the reviewed conception-related information was found to be inaccurate, the study did not explicitly identify or assess nutrition-related claims. Given that web searches are a primary source of fertility and conception information for many individuals^(11,12)^, understanding the accuracy of information available online is important to guide the development of future public health campaigns aimed at targeting misinformation.

With a clear shift towards the use of online resources and social media by people of reproductive age for health and nutrition information^(3)^, understanding the quality of online information and how the community perceives online nutrition fertility claims is needed for improved dissemination of scientific knowledge to consumers. This project aims to (1) explore and analyse current online preconception health and nutrition-related claims from Google, YouTube, OpenAI, Instagram and TikTok, (2) assess the identified online preconception health claims against a selection of current guidelines and (3) understand the perceived accuracy of a random sample of identified claims among reproductive-aged men and women living in the community.

## Methods

### Search strategy and data collection

This study involved a content analysis of online platforms, encompassing websites and social media dedicated to providing guidance on preconception nutrition and lifestyle behaviours for individuals of reproductive age. The intention of the search strategy was to employ lay terms and simple search methodologies. Over a 2-d period in May 2023, through an incognito Google Chrome browser, the terms ‘fertility nutrition’, ‘lifestyle changes when trying to conceive’ and ‘ways to increase fertility’ were searched on Google, the video-sharing platform YouTube and the conversational artificial intelligence (AI) platform OpenAI (see online supplementary material, Supplementary Fig. 1). The search terms were transformed into concise hashtags suitable for use on visually oriented platforms such as Instagram and TikTok with a newly created, generic account. The adapted hashtags included ‘#fertilitydiet’, ‘#TTClifestyle’ and ‘#increasefertility’. Search terms were decided through consultation between study authors, with the aim to simulate a population of individuals searching for fertility-related information. Video media across YouTube, TikTok and Instagram were transcribed verbatim prior to the extraction of claims. Media platforms such as Facebook, where claims were located within a private group, were not included in the study. From each of the three searches per platform, the top ten articles and posts were extracted, as most consumers are unlikely to view search results beyond the first page^(13)^.

### Content analysis

Thematic analysis was selected as the method for content analysis^(14)^. The content of the website or social media post was thoroughly read to identify any health claims, which were then extracted. A piece of text was identified as a claim if it was phrased as a statement or advice regarding behaviours, causes and potential methods used to impact fertility. Once extracted, inductive open coding was utilised to code the health claims using NVivo 12 Pro Plus. Each claim was assigned an alphanumeric identifiable code to link to the online or social media platform. Following the labelling of claims, they were grouped into recurring topics. This created a framework to systematically distribute content into appropriate topics for the remaining articles of information. Any topic unrelated to the aims was excluded; only claims related to nutrition, physical activity and general health topics (i.e. sleep, age and weight) were included. After discussing the topics, the first author developed the initial themes and categories, which were reviewed by senior authors and refined. Themes and categories were agreed upon by all authors. Once assigned to a theme and then category, claims were allotted back under their original platform (Google, YouTube, OpenAI, Instagram, TikTok). Duplicate claims were removed to ensure there were no repeats of a claim within each online or social media type.

### Assessing the evidence for the claim

The final list of 191 claims was analysed by a panel of three researchers with expert knowledge in nutrition, physiology and reproductive health. A 2022 systematic review of eleven Australian and International preconception guidelines was used to identify relevant guidelines, of which nine were used to compare against each claim^(15)^. Two guidelines present in the aforementioned systematic review were excluded from use by the expert panel. These guidelines had a limited scope, offering guidance on preconception care only for people living with human immunodeficient virus and Zika virus. A rubric for assessment of evidence was established by the study authors, whereby a claim present in the set of Australian or International preconception guidelines was considered the highest level of evidence. These included a claim reported in Royal Australian College of General Practitioners Guidelines^(16)^, Royal Australian and New Zealand College of Obstetricians and Gynaecologists^(17)^, South Australian Perinatal Practice Guidelines Preconception Advice^(18)^, Public Health Agency of Canada^(19)^, American College of Obstetricians and Gynecologists^(20)^, Federation of Obstetric and Gynecological Societies of India^(21)^, Centres for Disease Control and Prevention^(22)^, American Academy of Family Physicians (Positions Paper)^(23)^ and Recommendations for Preconception Counselling and Care^(24)^. The second highest level of evidence was judged against the most recent and comprehensive scoping review of observational studies assessing nutritional intake and female fertility outcomes, published in 2023^(25)^. The panel assessed each claim as being present in a preconception guideline (yes or no), present in the scoping review of female fertility (yes or no), having limited evidence to suggest an association or no association, having insufficient human evidence to support the claim or having no evidence to support the claim (see online supplementary material, Supplementary Fig. 2). A claim was considered to have limited evidence if it was indeed present in the scoping review of female fertility; however, definitive conclusions could not be made. A claim was considered to have insufficient evidence if, from a formative review of the literature, the expert panel could not identify human research but could identify some *in vitro* or animal evidence. The experts agreed upon the classification of each claim.

### Determining the community perception of claims

Following the content analysis and categorisation of claims, forty online claims were randomly selected (i.e. eight claims from each media source) for assessment by a sample of men and women (‘community’). The claims were randomly ordered to create a survey through REDCap, hosted by the University of Adelaide. The survey was distributed through the university and institute emails and newsletters and shared on social media platforms from October to November 2023. Inclusion criteria were aged 18–49 years and able to read and write in English. The survey was administered anonymously, participants provided informed consent, and participation was voluntary. Prior to undertaking the survey, each participant provided their gender (male, female or other), their date of birth and their occupation category (academic; health professional (fertility related); health professional (not fertility related); or neither an academic nor health professional). For pregnancy planning status, participants were instructed to select a single option that best described their current status from the following mutually exclusive choices: (1) not currently trying to conceive, (2) trying to conceive, (3) currently pregnant or (4) had a prior pregnancy. Each participant could select only one option, and these were treated as self-reported categorical descriptors, not objective reproductive statuses. This variable was not used in the analysis. For each claim, participants rated the likelihood of the claim being true using a 5-point Likert scale (1 = not at all likely, 2 = somewhat likely, 3 = unsure, 4 = quite likely, 5 = highly likely). A claim was considered likely to be true by the community if over 75 % of respondents rated the claim as greater than 4 on the Likert scale. Likewise, a claim was considered unlikely to be true if over 75 % of respondents rated the claim as less than 2 on the Likert scale.

### Data reporting

All data, including the content analysis, expert panel rating and online survey results, are summarised with raw numbers and percentages. Figures were created using BioRender^(26)^.

## Results

### Content analysis

Two primary themes were generated: *nutrition* and *lifestyle* (Table 1). Any claim with mention of food, nutrients, diet or supplements was categorised under the nutrition theme, and any claim related to an individual’s way of living or behaviours was categorised under the lifestyle theme. The nutrition theme contained 159 claims and was divided into three categories (*whole foods and their components*, *dietary patterns* and *supplements*). The lifestyle theme included thirty-two claims and was divided into three categories (*sleep and stress*, *physical activity* and *personal characteristics*). The category *whole foods and their components* contained the highest number of claims (*n* 64), while *physical activity* contained the least (*n* 5).

**Table 1.** All topics of claims included in each category and theme, including the number of claims in each category

Themes	Categories (number of claims)	Topics
Nutrition (159)	Dietary pattern (56)	Mediterranean, anti-inflammatory, high sugar, carnivore, dairy free, wholegrains, processed food, high fat
	Whole foods and their components (64)	Gluten, soy, alcohol, caffeine, trans fat, carbohydrates, antioxidants, fibre, phytoestrogens, cholesterol, specific foods
	Supplements (39)	Iron, coenzyme Q 10, omega 3, folic acid, iodine, vitamins A, C, D and E
Lifestyle (32)	Sleep and stress (13)	Stress reduction, sleep monitoring, anxiety
	Physical activity (5)	High intensity exercise, yoga, pilates, walking
	Personal characteristics (14)	Body weight, age

The YouTube platform contained the highest number of extracted claims (*n* 58), closely followed by Google (*n* 54 claims) (Table 2). Ninety-four percent of Instagram claims and 93 % of TikTok claims were present in the *nutrition* theme. OpenAI had the highest proportion of *lifestyle* themes when compared with other platforms, with 29 % (6/21) of claims from the *lifestyle* theme. All platforms had a high percentage of claims from the category *whole foods and their components* except for TikTok, with only 15 % (4/26) claims in this category. The social media platforms Instagram and TikTok had no claims in the category *physical activity*, and TikTok did not have any claims present in the category *personal characteristics*.

**Table 2.** Count and percentage of main themes and categories of claims found through the online searches on the platforms Google, YouTube, OpenAI, Instagram and TikTok

		Claim frequency and percentage of total claims by platform									
		Google *n 54 claims*		YouTube *n 58 claims*		OpenAI *n 21 claims*		Instagram *n 30 claims*		TikTok *n 28 claims*	
		*n*	%	*n*	%	*n*	%	*n*	%	*n*	%
Theme	Category										
Nutrition		**42**	**78 %**	**48**	**83 %**	**15**	**71 %**	**28**	**94 %**	**26**	**93 %**
	Dietary pattern	16	30 %	17	29 %	4	18 %	8	27 %	11	39 %
	Whole foods and their components	18	34 %	17	29 %	9	43 %	16	53 %	4	15 %
	Supplements	8	14 %	14	25 %	2	10 %	4	13 %	11	39 %
Lifestyle		**12**	**22 %**	**10**	**17 %**	**6**	**29 %**	**2**	**6 %**	**2**	**7 %**
	Sleep/stress	5	9 %	3	5 %	2	10 %	1	3 %	2	7 %
	Physical activity	2	4 %	2	3 %	1	5 %	0		0	
	Personal characteristics	5	9 %	5	9 %	3	14 %	1	3 %	0	

### Accuracy of claims

Five percent (10/191) of the identified claims are referred to in current preconception guidelines. These claims included statements relating to folic acid and iron supplementation, excess vitamin A consumption and healthy BMI ranges for conception. Two percent (4/191) of claims, while not present in a preconception guideline, were included in a recent scoping review of female fertility, with potential benefits identified for adherence to a Mediterranean diet and a diet low in trans-SFA. Six percent (11/191) of claims were deemed to have ‘limited evidence to suggest that there was no association’ between the claim and the reported health outcome, while 12 % (21/191) of claims were considered to have ‘limited evidence to suggest an association’ between the claim content and health outcome. Twenty-one percent (40/191) of claims were considered to have ‘insufficient evidence’, while 54 % (103/191) of claims were considered to have ‘no evidence for the health outcome’ (see online supplementary material, Supplementary Fig. 3).

**Fig. 1:**
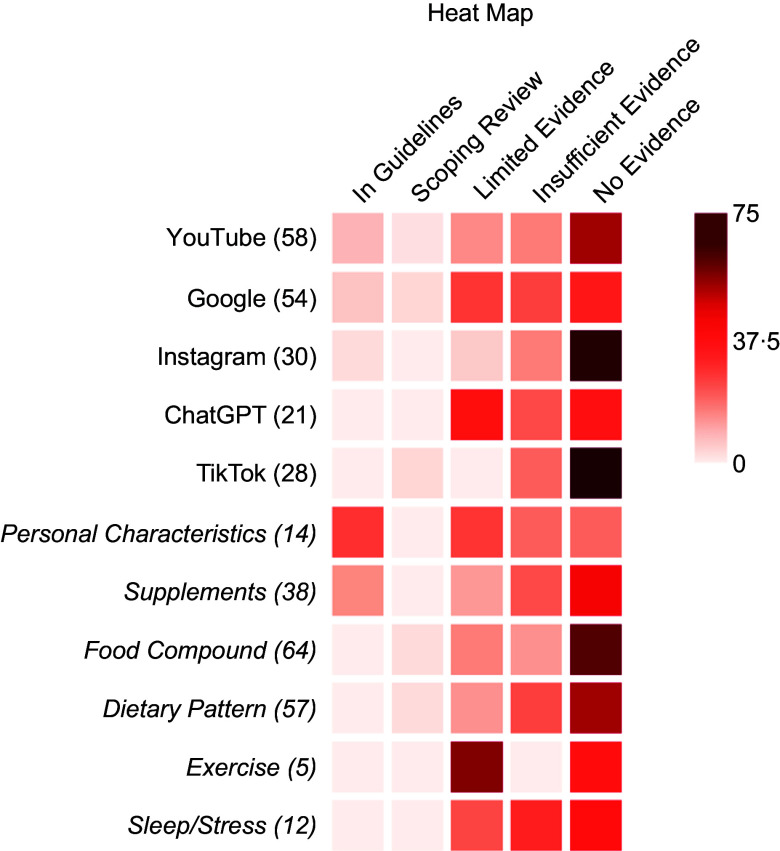
Heatmap illustrating the level of evidence supporting preconception health information across different online platforms and thematic categories. The rows represent the sources of information (*YouTube, Google, Instagram, OpenAI, TikTok*) and key preconception health topics (*personal characteristics, supplements, food compounds, dietary patterns, exercise, sleep/stress*), as well as the number of claims obtained from each source. Colour intensity corresponds to the percentage of claims from each platform or category within each level of evidence Source: Created in BioRender. Lush, K. (2025) https://BioRender.com/ruztax7.

Figure 1 reveals that a substantial percentage of preconception health information found online lacks strong scientific support, with many claims falling into the ‘insufficient evidence’ or ‘no evidence for the health outcome’ categories. TikTok and Instagram were found to have a higher percentage of claims with ‘no evidence for the health outcome’ than the other social media platforms (Fig. 1). The platforms YouTube, Google and Instagram contained claims that could be identified in preconception guidelines (Fig. 1), while only the categories *supplementation* and *personal characteristics* contained claims present in guidelines (Fig. 1). These claims were related to folic acid supplementation, iron supplementation, body weight and physical activity. A substantial portion of claims made about *whole foods and their components* and their impact on fertility had ‘no evidence for the health outcome’ stated in the claim (Fig. 1). No claims found through OpenAI or within the categories *sleep and stress* or *physical activity* were supported by high-level evidence, as none of the claims identified were present in preconception guidelines or the scoping review of female fertility.

### Community perception of health claims

Eighty participants completed the online survey, and their characteristics are reported in Table 3. Ninety-three percent of the survey participants were female, and the median age of the sample population was 33·9 (IQR 30·6, 37·2) years. Most participants (51·3 %) considered themselves to be neither a researcher nor a health professional, and 50 % of participants were not currently trying to conceive. Of the forty health claims presented, three were considered ‘likely to be true’, of which all three were deemed to have ‘insufficient evidence’ by the expert panel and one was considered to have ‘no evidence for the health outcome’ (Table 4). Three of the forty claims were considered ‘unlikely to be true’ by the community (score <2), of which the expert panel identified one to have ‘insufficient evidence’ to suggest an association and two to have ‘no evidence for the health outcome’ (Table 4). Of the forty claims presented to the community, the expert panel considered 17 claims to have ‘no evidence for the health outcome’; however, only two claims were identified as ‘unlikely to be true’ by the community.

**Table 3.** Demographic details of participants who completed the public survey of health claims

Gender	*n*	%
Female	75	93·8
Male	5	6·3
Age		
< 25 years	3	3·8
25–29 years	13	16·3
30–34 years	31	38·8
35–39 years	21	26·3
40–44 years	7	8·8
> 45 years	5	6·3
Self-reported profession		
Academic	12	15·0
Health professional (fertility related)	1	1·3
Health professional (other)	26	32·5
Neither researcher nor health professional	41	51·3
Pregnancy planning status		
Not currently trying to conceive	40	50·0
Actively trying to conceive	8	10·0
Currently pregnant	8	10·0
Prior pregnancy	24	30·0

**Table 4.** Claims rated by the community (*n* 80) as likely (>4) or unlikely (<2) to be true and the expert panel rating of each claim

Claim	Expert rating			Community assessment of claim^*^		*n* (%) of respondents who rated each claim as unlikely to likely to be true			
	In guidelines	Insufficient evidence	No evidence	Unlikely	Likely	Unlikely		Likely	
						*n*	%	*n*	%
Aiming for a healthy weight and BMI is beneficial while trying to conceive.	✓			Survey responses did not indicate that the community considered either claim to be likely or unlikely to be true.		44/80	55 %	23/80	28.75 %
Engaging in moderate exercise has been associated with improved ovulation and a shorter time to pregnancy. However, excessive exercise negatively impacts fertility.	✓						16/80	20 %	55/80	72.5 %
Regular exercise and good quality sleep will help reduce stress, which will promote regular ovulation and optimal egg health.		✓			✓	6/80	7.5%	74/80	92.5%
Fruit and berries are high in antioxidants, vitamin C and folic acid which promotes healthy foetal growth after conception.		✓			✓	12/80	15%	68/80	85%
Avoid alcohol before trying to conceive. Alcohol impacts ovulation and causes genetic disorders in the oocyte.		✓			✓	20/80	25%	60/80	75%
Processed soy is inflammatory and decreases fertility.		✓		✓		61/80	76.25 %	19/80	23.75 %
Gluten causes inflammation in the body which inhibits healthy egg formation and ovulation.			✓	✓		64/80	80 %	16/80	20 %
Consuming 1 cup of bone broth daily when trying to conceive over the age of 35 will health the gut lining and help absorb more nutrients that are critical for pregnancy.			✓	✓		60/80	75 %	20/80	25 %

* ≥75 % of the community rated the claim as <2 or >4 on the Likert scale when completing the survey, where <2 was considered unlikely to be true and >4 was considered likely to be true. Two claims, which were present in preconception guidelines, did not receive a high enough percentage of respondents rating the claim as >4 to be considered likely to be true by the community.

## Discussion

Our analysis highlights a significant gap between publicly shared nutrition and lifestyle claims and established preconception guidelines. While only a small proportion of identified claims aligned with existing recommendations, the majority lacked sufficient supporting evidence for the stated health outcomes. Notably, some claims that were considered likely to be true by the public were not reflected in preconception guidelines, suggesting a disconnect between community perception and evidence-based recommendations. This underscores the need for clearer public health communication and improved integration of emerging evidence into preconception care guidance.

With over six million users between the ages of 18–45, social media platforms can be a valuable way to create behavioural change within the community and to spread information^(27)^. All five platforms included in this study contained claims that were considered to have ‘no evidence for the health outcome’; however, TikTok (75 %) and Instagram (73 %) contained a higher percentage of ‘no evidence’ claims than the other media platforms (Google, YouTube and OpenAI, all <60 %). This is interesting given that TikTok is growing in popularity, with users spending upwards of 1 h/d on the platform^(28)^. As of 2024, 69 and 62 % of respective TikTok^(29)^ and Instagram users^(30)^ are aged between 18 and 34 years, suggesting that a high proportion of users on TikTok and Instagram are of reproductive age. As such, the information presented through social media platforms has the potential to impact preconception behaviours among this age group. Accessing reliable and consistent information is often perceived to be a barrier to implementing preconception nutrition and lifestyle changes^(31)^. These barriers may be more pronounced in vulnerable populations, such as those experiencing socio-economic disadvantage or having lower levels of education and health literacy^(31)^. However, it is important to distinguish between the ability to access and evaluate reliable information and the likelihood of acting on information encountered online. Research indicates that individuals with lower health literacy may be particularly influenced by the persuasive techniques and relatable narratives found on social media platforms such as Instagram and TikTok^(32)^. In these cases, behaviour change often occurs not because the information is reliable, but because it is accessible, engaging and presented by trusted content creators. This highlights a critical concern: while these platforms can promote behaviour change, they may also disseminate misleading or lower-quality information. Therefore, ensuring that social media content is accurate, evidence-based and accessible is particularly important for reaching and positively influencing populations with low health literacy.

OpenAI was the only platform where none of the claims presented were in the guidelines or scoping review of female fertility. Emerging research suggests AI will become an integral part of healthcare; however, challenges related to privacy, bias and the need for human expertise in healthcare should be addressed before the broader implementation of AI as a health tool^(33)^. AI has the advantage of synthesising information from multiple online sources to assist consumers from having to deduce their own conclusions when comparing information online. As a source of information, research suggests that AI is reasonably accurate^(34,35)^; however, it has been shown to become confused when provided with an overwhelming volume of information^(36)^. OpenAI provided information on which 8/21 (38 %) claims had ‘no evidence for the health outcome’. This suggests that AI needs further training to distinguish and provide accurate preconception information. The operations of AI rely on the information available online. Given there is little accurate information for AI to work with regarding preconception nutrition, vague or unsupported answers may be given to those seeking advice.

Two claims present in the online survey were readily available in preconception guidelines; however, neither was rated as ‘likely to be true’ by the community. These claims included ‘achieving a healthy weight and BMI is beneficial while trying to conceive’ and ‘engaging in moderate exercise is beneficial before pregnancy and can lead to healthy pregnancy outcomes’. All Australian and International preconception guidelines have stated that aiming for a healthy body weight prior to conception is beneficial^(16–24)^. However, no studies to date have examined the use of preconception guidelines by the community; thus, it is unknown if the community is aware of the recommendations made in such guidelines. There is potential that the community did not rate body weight-related claims as ‘likely to be true’ due to the recent emergence of body weight messaging across social media and online, where weight neutrality and body positivity movements gain traction^(37)^. Conversely, the participants surveyed seemingly did not consider the weight of significance when trying to conceive. This is notable as weight stigma, the widespread, stereotypical and harmful belief that being of a higher weight is unhealthy, has been shown to adversely affect fertility outcomes^(38)^. Efforts to promote healthy nutrition and lifestyle behaviours in a manner that is not stigmatising to those of a higher body weight may increase the uptake of preconception healthcare and positively impact the uptake of healthcare advice^(38)^. This will contribute to improved pregnancy outcomes and positively influence long-term health for both the mother and child.

No claims within the category *sleep and stress* were identified in preconception guidelines, despite this category representing nearly half of the claims within the *lifestyle* theme. Interestingly, five Australian and International preconception guidelines mention *physical activity* ^(16–19,21)^ for weight loss or weight management rather than a tool for improving health. In contrast, the most recent Australian pregnancy guidelines, which were updated in 2023, advocate for the inclusion of physical activity throughout pregnancy to lower the risk of complications and promote a healthy pregnancy^(39)^. These guidelines provide details regarding duration, intensity and type of physical activity to participate in during pregnancy, in addition to suggestions for activities to avoid. The discord between the absence of lifestyle behaviours in preconception guidelines and the high number of lifestyle-related claims identified in this search highlights a need for more high-quality research to be conducted to establish the impacts of pre-pregnancy lifestyle factors on conception and pregnancy, as well as for the expansion of guidelines to include a more holistic approach to preconception health as relevant high-level evidence emerges.

Three claims that were rated as ‘likely to be true’ by the community were deemed by the expert panel to have ‘limited’ or ‘insufficient’ evidence. When assessing the quality of claims, a common practice of juxtaposing a factually correct statement with a health outcome that lacked significant evidence was observed. For example, ‘avoid alcohol before trying to conceive. Alcohol impacts ovulation and causes genetic disorders in the oocytes’. While the National Health and Medical Research Council^(40)^ and preconception guidelines recommend reducing or abstaining from alcohol prior to pregnancy, ovulation and disruption of genetic material in oocytes are not considered the reasons for the avoidance. Instead, foetal alcohol spectrum disorders are evidenced for this recommendation in preconception guidelines^(16–24)^. If a claim was structured in this way, it was considered to have ‘no’ or ‘limited evidence’ by the expert panel, despite the former fragment of the statement being correct.

Correcting potential harm caused by online misinformation is a key goal of public health campaigns. This is demonstrated through the preconception guidelines^(16–24)^, the National Health and Medical Research Council in Australia^(40)^ and the WHO^(41)^, which all recommend, for example, abstinence from alcohol prior to conception in an effort to reduce the risk of foetal alcohol spectrum disorder, cognitive disabilities and birth defects^(42)^. Based on evidence, the public health campaign in Western Australia, ‘One Drink’, which promotes abstinence from alcohol prior to and during pregnancy^(43)^, was well received and considered acceptable by target audiences^(44)^. Exposure to the public health campaign created favourable intentions to change alcohol related behaviours^(43)^. However, the view of alcohol avoidance when trying to conceive has not been echoed across media platforms, with an Instagram claim stating that ‘four standard drinks per week is safe while trying to conceive’. One study suggested that social media is a vital part of successful public health campaigns^(45)^. The present study demonstrates a clear lack of high-level evidence utilised across social media platforms, despite professional organisations publishing clear recommendations regarding preconception alcohol use. As social media platforms heavily impact consumer knowledge base^(46)^, the incorporation of preconception guidelines into targeted social media public health campaigns would assist in disseminating high-level evidence to women and men accessing this information online. Engaging with consumers to better understand their internet use behaviours when accessing fertility-related information would facilitate effective strategies to target misinformation online.

### Strengths and limitations

This study had several key strengths. The chosen methodology, including searching three separate terms across five platforms, allowed us to simulate the consumer experience when exploring preconception information-seeking behaviours. This approach identified a wide breadth of claims encompassing nutrition and lifestyle behaviours and created a search environment that could be viewed by health professionals, family members or those trying to conceive. Another strength includes the use of video platforms that were searched in this study, with YouTube, TikTok and Instagram reels available for assessment. This allowed us to capture the most popular media platforms frequently utilised by a reproductive-aged population. In addition to research institutions, social media platforms were used to distribute the community survey. This facilitated an understanding of the community perceptions of health claims among a sample of reproductive-aged people living in Australia.

There are also limitations to consider. The community survey did not employ a formal sample size and therefore represents a convenience sample. This limits the generalisability of the findings and may mean the results do not capture the full diversity of the broader population. Nevertheless, our sample is larger than those used in previous Australian-based studies assessing product content claims, which included twenty-six^(47)^ and thirty-six^(48)^ men and women, respectively. Despite this limitation, our sample offers valuable insight into how the community perceives fertility-related nutrition and lifestyle claims. To reduce participant burden, we presented participants with a smaller sample of claims, which limits our understanding of how the broader community may understand and interpret all categories of claims. Furthermore, not all social media platforms were screened, including media platforms with closed groups. As such, the balance of claims available through online platforms may be different from that observed here. Additionally, our search was conducted on newly created social media profiles using web browsers in incognito mode. This approach differs from the experience of individuals routinely seeking preconception health information, as engagement with content can influence the algorithm to recommend additional profiles and posts. As a result, the likelihood of repeated exposure to unevidenced claims may be higher for regular users. We searched English platforms alone, so the information may not be directly translatable to non-English speaking individuals. We acknowledge the use of binary terms when recording gender identity and how this may have influenced responses. Sex at birth was not required for the purpose of this study as it explores social and cultural concepts, so the term gender was used to allow respondents to self-identify for the survey. Allowing further options for participants to indicate non-binary gender identities or gender diverse experiences would have provided a more inclusive understanding of perspectives on nutrition-related fertility claims and better captured the diversity of lived experiences they may hold.

## Conclusions

There is a wealth of inaccurate information across social media platforms regarding preconception nutrition and lifestyle behaviours, with many claims lacking support from high-level evidence. Preconception fertility-related health claims that were routinely present in Australian and International preconception guidelines were not perceived by the community as likely to be true. High-level evidence for preconception health needs to be established and disseminated, and targeted public health campaigns via social media would assist in improving the awareness of preconception guidelines and their content. Despite minimal representation in preconception guidelines, many claims authored online centre on lifestyle behaviours, suggesting there is an interest in improving overall preconception health. Further research assessing preconception health, nutrition and lifestyle behaviours is needed to contribute and strengthen the evidence base for peri-conception recommendations.

## Supporting information

Lush et al. supplementary material 1 Lush et al. supplementary material

Lush et al. supplementary material 2 Lush et al. supplementary material

Lush et al. supplementary material 3 Lush et al. supplementary material

## Supplementary material

For supplementary material accompanying this paper, visit https://doi.org/10.1017/S1368980025100876.

## References

[1] Song H, Omori K, Kim J et al. (2016) Trusting social media as a source of health information: online surveys comparing the United States, Korea, and Hong Kong. J Med Internet Res 18, e4193. 10.2196/jmir.4193 PMC4810010 26976273

[2] Skouteris H & Savaglio M (2021) The use of social media for preconception information and pregnancy planning among young women. J Clin Med 10, 1892. 33925520 10.3390/jcm10091892 PMC8123806

[3] Orizio G, Schulz P, Gasparotti C et al. (2010) The world of e-patients: a content analysis of online social networks focusing on diseases. Telemed E-Health 16, 1060–1066. 10.1089/tmj.2010.0085 21070131

[4] Cash T, Desbrow B, Leveritt M et al. (2014) Utilization and preference of nutrition information sources in Australia. Health Expect 18, 2288–2295. 24798108 10.1111/hex.12198 PMC5810644

[5] Lush KR, Hutchison AT, Pacella-Ince L et al. (2025) Understanding the experiences of women seeking preconception health information. Women’s Reprod Health 12, 1, 100–117.

[6] Sayakhot P & Carolan-Olah M (2016) Internet use by pregnant women seeking pregnancy-related information: a systematic review. BMC Pregnancy Childbirth 16, 65. 27021727 10.1186/s12884-016-0856-5 PMC4810511

[7] Denniss E, Lindberg R & McNaughton SA (2023) Quality and accuracy of online nutrition-related information: a systematic review of content analysis studies. Public Health Nutr 26, 1345–1357. 37138366 10.1017/S1368980023000873 PMC10346027

[8] Denniss E, Lindberg R, Marchese LE et al. (2024) #Fail: the quality and accuracy of nutrition-related information by influential Australian Instagram accounts. Int J Behav Nutr Phys Act 21, 6. 38355567 10.1186/s12966-024-01565-y PMC10865719

[9] Peyser A, Goldstein L, Mullin C et al. (2021) Fertility education: what’s trending on Instagram. Fertil Res Pract 7, 3. 33461628 10.1186/s40738-021-00095-6 PMC7812639

[10] Kedzior SGE, Bianco-Miotto T, Breen J et al. (2019) It takes a community to conceive: an analysis of the scope, nature and accuracy of online sources of health information for couples trying to conceive. Reprod Biomed Soc Online 9, 48–63. 32021914 10.1016/j.rbms.2019.08.004 PMC6994282

[11] Hammarberg K, Zosel R, Comoy C et al. (2016) Fertility-related knowledge and information-seeking behaviour among people of reproductive age: a qualitative study. Hum Fertil 20, 88–95. 10.1080/14647273.2016.1245447 27778517

[12] Broughton DE, Schelble A, Cipolla K et al. (2018) Social media in the REI clinic: what do patients want? J Assist Reprod Genet 35, 1259–1263. 29766400 10.1007/s10815-018-1189-2 PMC6063835

[13] Morahan-Martin JM (2004) How Internet users find, evaluate, and use online health information: a cross-cultural review. Cyberpsychol Behav 7, 497–510. 15667044 10.1089/cpb.2004.7.497

[14] Braun V & Clarke V (2006) Using thematic analysis in psychology. Qual Res Psychol 3, 77–101.

[15] Dorney E, Boyle JA, Walker R et al. (2022) A systematic review of clinical guidelines for preconception care. Semin Reprod Med 40, 157–169. 35576970 10.1055/s-0042-1748190

[16] RACGP (2018) Royal Australian College of General Practitioners Guidelines for Preventive Activities in General Practice. East Melbourne: RACGP.

[17] RANZCOG (2021) RANZCOG Best Practice Statement Pre-pregnancy Counselling. Melbourne: RANZCOG.

[18] Government of South Australia (2015) Preconception Advice Clinical Guideline. Adelaide: SA Health. Available at https://www.sahealth.sa.gov.au/wps/wcm/connect/1f11de804eed8cb5afbeaf6a7ac0d6e4/Preconception+Advice_Sept2015.pdf?MOD=AJPERES&CACHEID=ROOTWORKSPACE-1f11de804eed8cb5afbeaf6a7ac0d6e4-ocRgEPC https://www.sahealth.sa.gov.au/wps/wcm/connect/1f11de804eed8cb5afbeaf6a7ac0d6e4/Preconception+Advice_Sept2015.pdf?MOD=AJPERES&CACHEID=ROOTWORKSPACE-1f11de804eed8cb5afbeaf6a7ac0d6e4-ocRgEPC (accessed 19 November 2023).

[19] Shaw E, Barney L, Di Meglio G et al. (2021) Chapter 2: Preconception Care. Ottawa: Government of Canada. Available at https://www.canada.ca/en/public-health/services/publications/healthy-living/maternity-newborn-care-guidelines-chapter-2.html (accessed 18 November 2023).

[20] ACOG (2019) ACOG Committee Opinion no. 762: prepregnancy counseling. Obstet Gynecol 133, e78–89. 30575679 10.1097/AOG.0000000000003013

[21] Federation of Obstetric and Gynaecological Societies of India (n.d.) Good Clinical Practice Recommendations on Preconception Care. Available at https://www.fogsi.org/gcpr-preconception-care/ (accessed October 2023).

[22] Centers for Disease Control and Prevention (2023) Planning for Pregnancy. Available at https://www.cdc.gov/preconception/planning.html (accessed October 2023).

[23] American Academy of Family Physicians (2022) Preconception Care (Position Paper). Available at https://www.aafp.org/about/policies/all/preconception-care.html (accessed October 2023).

[24] Farahi N & Zolotor A (2013) Recommendations for preconception counseling and care. Am Fam Physician 88, 499–506. 24364570

[25] Alesi S, Habibi N, Silva TR et al. (2023) Assessing the influence of preconception diet on female fertility: a systematic scoping review of observational studies. Hum Reprod Update 29, 811–828. 37467045 10.1093/humupd/dmad018 PMC10663051

[26] BioRender (n.d.) BioRender. Available at https://BioRender.com (accessed May 2025).

[27] Evans WD, Abroms LC, Broniatowski D et al. (2022) Digital media for behavior change: review of an emerging field of study. Int J Environ Res Public Health 19, 9129. 35897494 10.3390/ijerph19159129 PMC9331057

[28] Kemp S (2024) *Digital 2024: Global Overview Report*. Data Reportal; published online January 31. Available at https://datareportal.com/reports/digital-2024-global-overview-report (accessed February 2024).

[29] Oberlo (2024) TikTok Age Demographics. Available at https://www.oberlo.com/statistics/tiktok-age-demographics (accessed April 2024).

[30] Oberlo (2024) Instagram Age Demographics. Available at https://www.oberlo.com/statistics/instagram-age-demographics#:~:text=According%20to%20recent%20research%20on,which%2030.6%25%20of%20Instagrammers%20belong (accessed April 2024).

[31] Khan NN, Boyle JA, Lang AY et al. (2019) Preconception health attitudes and behaviours of women: a qualitative investigation. Nutrients 11, 1490. 31261954 10.3390/nu11071490 PMC6682867

[32] Zhu Z, Liu S & Zhang R (2023) Examining the persuasive effects of health communication in short videos: systematic review. J Med Internet Res 25, e48508. 37831488 10.2196/48508 PMC10612001

[33] Alowais SA, Alghamdi SS, Alsuhebany N et al. (2023) Revolutionizing healthcare: the role of artificial intelligence in clinical practice. BMC Med Educ 23, 689. 37740191 10.1186/s12909-023-04698-z PMC10517477

[34] Kirk D, Van Eijnatten E & Camps G (2023) Comparison of answers between ChatGPT and human dieticians to common nutrition questions. J Nutr Metab 2023, 1–9. 10.1155/2023/5548684 PMC10645493 38025546

[35] Beilby K & Hammarberg K (2024) ChatGPT: a reliable fertility decision-making tool? Hum Reprod 39, 443–447. 38199794 10.1093/humrep/dead272 PMC10905498

[36] Koopman B & Zuccon G (2023) Dr ChatGPT tell me what I want to hear: how different prompts impact health answer correctness. Proc Conf Empir Methods Nat Lang Process 2023, 15012–15022.

[37] Clark O, Lee MM, Jingree ML et al. (2021) Weight stigma and social media: evidence and public health solutions. Front Nutr 8, 739056. 34869519 10.3389/fnut.2021.739056 PMC8632711

[38] Hill B & Incollingo Rodriguez AC (2020) Weight stigma across the preconception, pregnancy, and postpartum periods: a narrative review and conceptual model. Semin Reprod Med 38, 414–422. 33728621 10.1055/s-0041-1723775

[39] Australian Government Department of Health and Aged Care (2023) Guidelines for Physical Activity During Pregnancy. Canberra: Australian Government Department of Health and Aged Care.

[40] National Health and Medical Research Council (2020) Australian Guidelines to Reduce Health Risks from Drinking Alcohol. Canberra: NHMRC.

[41] World Health Organization (2006) Framework for Alcohol Policy in the WHO European Region. Copenhagen: WHO Regional Office for Europe.

[42] Dejong K, Olyaei A & Lo JO (2019) Alcohol use in pregnancy. Clin Obstet Gynecol 62, 142–155. 30575614 10.1097/GRF.0000000000000414 PMC7061927

[43] Pettigrew S, Booth L, McCausland T et al. (2023) Evaluation outcomes of a Western Australian campaign designed to reduce alcohol use in pregnancy. Aust N Z J Public Health 47, 100102. 37993367 10.1016/j.anzjph.2023.100102

[44] Pettigrew S, Booth L, McCausland T et al. (2023) Evaluation outcomes of an alcohol and pregnancy campaign targeting multiple audiences. Drug Alcohol Rev 42, 36–45. 36066382 10.1111/dar.13541 PMC10087540

[45] Golechha M (2016) Health promotion methods for smoking prevention and cessation: a comprehensive review of effectiveness and the way forward. Int J Prev Med 7, 6. 26941908 10.4103/2008-7802.173797 PMC4755211

[46] Ghahramani A, De Courten M & Prokofieva M (2022) The potential of social media in health promotion beyond creating awareness: an integrative review. BMC Public Health 22, 2402. 36544121 10.1186/s12889-022-14885-0 PMC9770563

[47] Thompson B, McMahon A, Watson WL, et al. (2004) Consumer perceptions of nutrient content claims in Australia: A qualitative study. J Hum Nutr Diet; 37: 168–81. 10.1111/jhn.13241 37752748

[48] Chan C, Patch C & Williams P (2005) Australian consumers are sceptical about but influenced by claims about fat on food labels. Eur J Clin Nutr 59: 148–151. 15305180 10.1038/sj.ejcn.1602038

