# Supplementary material for: Evaluating the quality of online fertility nutrition claims
Source: Public Health Nutr. 2025 Aug 12;28(1):e151. doi: 10.1017/S1368980025100876 (PMC12516624; doi:10.1017/S1368980025100876)
Supplement: Lush et al. supplementary material 1 — Lush et al. supplementary material [file S1368980025100876sup001.docx]

|  | **Search Terms** |
| --- | --- |
| **Google, YouTube, Chat GPT** | Fertility Nutrition; Lifestyle Changes When Trying to Conceive; Ways to Increase Fertility |
| **Instagram, TikTok** | #fertilitydiet; #TTClifestyle; #increasefertility |

Supplementary File 1: Search terms used across platforms
