# Supplementary material for: Evaluating the quality of online fertility nutrition claims
Source: Public Health Nutr. 2025 Aug 12;28(1):e151. doi: 10.1017/S1368980025100876 (PMC12516624; doi:10.1017/S1368980025100876)

**Supplementary File 2:** Hierarchy of evidence used by the expert panel to assess online fertility health claims.


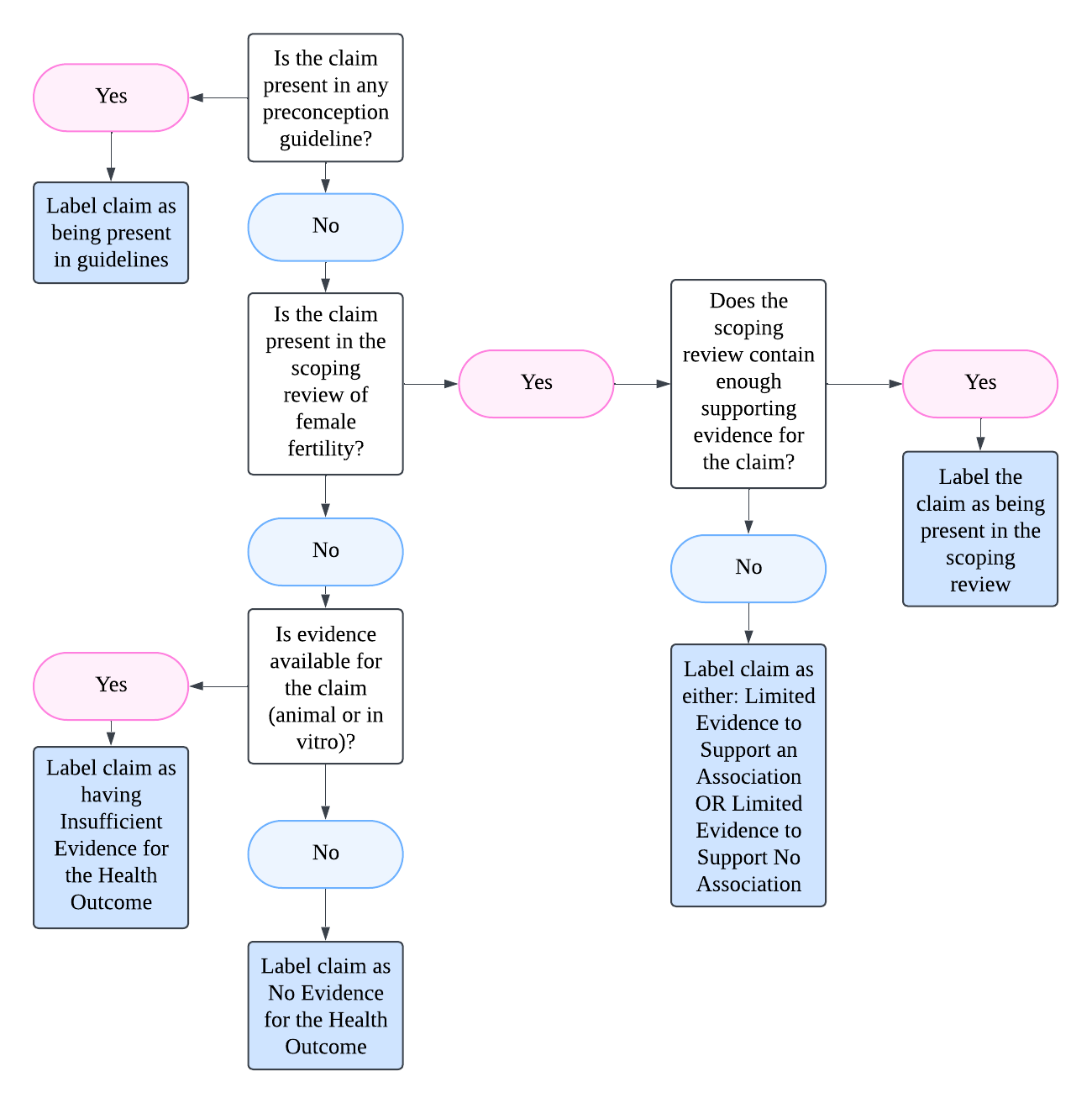

Supplement: Lush et al. supplementary material 2 — Lush et al. supplementary material [file S1368980025100876sup002.docx]
