# Supplementary material for: Evaluating the quality of online fertility nutrition claims
Source: Public Health Nutr. 2025 Aug 12;28(1):e151. doi: 10.1017/S1368980025100876 (PMC12516624; doi:10.1017/S1368980025100876)
Supplement: Lush et al. supplementary material 3 — Lush et al. supplementary material [file S1368980025100876sup003.docx]

**Supplementary File 3:** Fertility health claim and analysis by expert panel.

| **Fertility Nutrition Health Claim** | **Expert Analysis of Claim** |
| --- | --- |
| There is a higher incidence of ovulatory disorder in those who consumed more trans fats, sugar from carbohydrates, and animal proteins. | Limited Evidence to Suggest NO Association |
| Gluten causes inflammation in the body which inhibits healthy egg formation and ovulation | No evidence for the health outcome |
| Folic acid is the only extra supplement everyone needs if you’re trying to get pregnant. | In Guidelines |
| Too much caffeine can also increase your risk of miscarriage. | Limited Evidence to Suggest AN Association |
| Sugar makes your ovaries cystic and impacts ovulation, increasing your time to pregnancy | Insufficient Evidence to Suggest AN Association |
| They get a bad rap from cholesterol, but the yolk has excellent stores of protein and choline, a vitamin that helps develop brain function in babies | Limited Evidence to Suggest NO Association |
| Avoid the night shift, if possible. Regularly working the night shift might put you at higher risk of infertility, possibly by affecting hormone production. If you do work the night shift, try to get enough sleep when you're not working. | Limited Evidence to Suggest AN Association |
| Don't smoke. Tobacco use is associated with lower fertility. Smoking ages your ovaries and depletes your eggs prematurely. | Insufficient Evidence to Suggest AN Association |
| A woman who is overweight can take twice as long to become pregnant as a woman whose body mass index (BMI) is considered normal weight, | Insufficient Evidence to Suggest AN Association |
| A high intake of low-fat dairy has been shown to raise the risk of ovulatory infertility compared to high-fat dairy. | Limited Evidence to Suggest NO Association |
| If you're having continued trouble conceiving, you may want to consider limiting dairy from your fertility diet plan altogether. | No evidence for the health outcome |
| The Mediterranean diet has lots of fresh vegetables and fruit, whole grains, legumes, fish and olive oil and very little red meat. Research suggests it can also boost fertility and reduce the risk of gestational diabetes | Insufficient Evidence to Suggest AN Association |
| Eat more fibre, as it helps you keep your blood sugar balanced and can reduce excess hormones in the body that cause infertility | No evidence for the health outcome |
| Consuming alcohol has been linked to taking longer to get pregnant and other infertility issues. Consider limiting alcohol as much as possible where you can. | No evidence for the health outcome |
| Artificial sweeteners are stressors on your system; they create a cortisol response, which inhibits ovulation, increasing your time to pregnancy | No evidence for the health outcome |
| A sedentary lifestyle has been associated with higher risk of infertility. | Limited Evidence to Suggest AN Association |
| Fruit and berries are high in antioxidants, Vitamin C and folic acid which promotes healthy foetal growth after conception. | Insufficient Evidence to Suggest AN Association |
| Studies show that eating one cup of boiled asparagus will provide you with over 60% of your daily recommended value of folic acid, fulfill your daily vitamin K value, and strengthen your reproductive health with more than 20% of vitamins A, C, and B. | No evidence for the health outcome |
| Eliminate trans fats from your diet, too, as consuming too much has been linked to infertility. | In Scoping Review |
| 30% of infertility has been associated with weight extremes. Infertility rates are 3 times higher in obese women. | Limited Evidence to Suggest AN Association |
| Women who work at night may disrupt their circadian rhythm, which can cause hormonal imbalances that make pregnancy more difficult to achieve | Limited Evidence to Suggest AN Association |
| Aiming for a healthy weight and BMI is beneficial while trying to conceive | In Guidelines |
| St. John's wort, echinacea purpurea, and ginkgo biloba made the eggs impossible or difficult to fertilize, changed the genetic material in sperm, and reduced a sperm's viability. | No evidence for the health outcome |
| If consuming dairy products try and consume non-fat products and avoid ice-cream as this will negatively impact your fertility | No evidence for the health outcome |
| Watermelon and asparagus, in addition to other raw fruits and vegetables, give the body a rich supply of glutathione, which is important for egg quality | No evidence for the health outcome |
| An eating pattern rich in iron that comes from vegetables and supplements may lower the risk of ovulatory infertility | Limited Evidence to Suggest AN Association |
| Kale is another powerhouse vegetable because it contains elements necessary for estrogen metabolism, which promotes good fertility health | No evidence for the health outcome |
| A higher folate intake was associated with higher rates of implantation, clinical pregnancy, and live birth. | Insufficient evidence to suggest an association |
| Exercise has many benefits for your health, including increased fertility. | Limited Evidence to Suggest NO Association |
| Stress can suppress fertility. | Limited Evidence to Suggest AN Association |
| A study of women with endometriosis found that taking bee propolis twice a day resulted in a 40 percent greater chance of becoming pregnant after 9 months. | No Evidence for the health outcome |
| Eating more calories at breakfast and fewer at your evening meal can improve fertility. | No evidence for the health outcome |
| The Mediterranean diet which is high in antioxidants has been shown to be one of the healthiest in the world for general health and hence fertility. | In Scoping Review |
| Consuming a certain quantity of monounsaturated fats in the form of avocados during the IVF cycle increased the success rate by 300% | No evidence for the health outcome |
| Consumption of sugar-sweetened beverages (especially sodas or energy drinks) was linked to lower fertility for men and women, while drinking diet soda and fruit juice had no effect. | Limited Evidence to Suggest AN Association |
| Couples eating more seafood were pregnant sooner than those rarely eating seafood. | Insufficient Evidence to Suggest AN Association |
| Avoid excessive vitamins before conception. Too much vitamin A, for example, can be bad for a developing foetus. | In Guidelines |
| If you are overweight, losing between 5% and 10% of your weight can jump-start ovulation, according to the research. | In Guidelines |
| To increase fertility, try to incorporate more complex ("slow") carbs and limit highly processed ones. | No Evidence for the health outcome |
| Phytoestrogens are known to negatively impact fertility in humans and other animals. | No Evidence for the health outcome |
| Liver is a premium source of vitamins C, D, E, Co-Q10, Zinc, Folate, and fat – all of which have been shown to play crucial roles in male and female fertility. | No evidence for the health outcome |
| Choline is known to reduce the risk of some birth defects. | Insufficient Evidence to Suggest AN Association |
| Iodine deficiencies have been linked to reduced fertility. | Limited Evidence to Suggest AN Association |
| Selenium is an antioxidant that supports healthy follicles in the ovaries, which develop and release eggs. | Insufficient Evidence to Suggest AN Association |
| Putrescine is also believed to improve egg health, especially in women 35 and older. | Limited Evidence to Suggest AN Association |
| Pomegranates are rich in antioxidants that are rumoured to assist with fertility. | Limited Evidence to Suggest NO Association |
| Excess body fat, particularly around the abdomen, can lead to hormonal imbalances, such as increased insulin levels and higher levels of estrogen. This can affect your time to a pregnancy. | Insufficient Evidence to Suggest AN Association |
| Women with a healthy weight have higher success rates with IVF and other assisted reproductive techniques. | Limited Evidence to Suggest AN Association |
| Low-fat dairy products may be associated with improved fertility outcomes | Limited Evidence to Suggest NO Association |
| Omega-3s have been associated with improved egg quality in women. | Limited Evidence to Suggest AN Association |
| CoQ10 is involved in the energy production process within the cells, including the eggs. CoQ10 may contribute to the optimal development and maturation of eggs. | Limited Evidence to Suggest AN Association |
| Engaging in moderate exercise has been associated with improved ovulation and a shorter time to pregnancy. However, excessive exercise negatively impacts fertility. | In Guidelines |
| High levels of stress can interfere with reproductive function and hormonal balance, making it more challenging to conceive. | No Evidence for the health outcome |
| Lack of quality sleep has been associated with decreased egg quality in women. | Insufficient Evidence to Suggest AN Association |
| High caffeine consumption may be associated with longer time to conceive and a higher risk of miscarriage. Additionally, there is some evidence to suggest that caffeine may interfere with the process of egg maturation and implantation in the uterus | Limited Evidence to Suggest AN Association |
| Many processed foods are associated with higher levels of inflammation and oxidative stress in the body. Chronic inflammation and increased oxidative stress can disrupt hormonal balance and reproductive function, potentially affecting fertility. | Insufficient Evidence to Suggest AN Association |
| Avoid alcohol before trying to conceive. Alcohol impacts ovulation and causes genetic disorders in the oocyte. | No evidence for the health outcome |
| Resveratrol is a compound found in certain fruits (such as grapes and berries) and red wine. It has antioxidant and anti-inflammatory properties improves egg quality and fertility outcomes. | No evidence for the health outcome |
| Antioxidants help protect cells, including eggs, from oxidative damage. Foods rich in antioxidants include berries, leafy greens, colourful vegetables, nuts, seeds, and whole grains. | No evidence for the health outcome |
| A high-fibre diet promotes a healthy gut microbiome, which plays a role in nutrient absorption, hormone metabolism, and overall immune function. A balanced gut microbiome is associated with improved reproductive health. | Insufficient Evidence to Suggest AN Association |
| Fibre aids in the elimination of excess hormones from the body, particularly estrogen. This can be beneficial for women who have infertility and help them fall pregnant faster. | No evidence for the health outcome |
| Omega-3s contribute to the development of a healthy uterine lining (endometrium) which will increase implantation. | No Evidence for the health outcome |
| Fish oil supplements can provide higher concentrations of omega-3 fatty acids compared to consuming fish alone, which makes it better for fertility and an essential supplement when trying to conceive. | No evidence for the health outcome |
| Monounsaturated fats, found in foods such as avocados, olive oil, and nuts, have been associated with improved fertility in both men and women. | Limited Evidence to Suggest AN Association |
| Trans fats, found in partially hydrogenated oils often used in processed and fried foods, have been associated with fertility problems. | Limited Evidence to Suggest AN Association |
| Eating 14 serves of vegetables per week reduces your risk of miscarriage | Insufficient Evidence to Suggest AN Association |
| Cayenne pepper helps boost female fertility | No evidence for the health outcome |
| Ginger protects eggs from oxidative stress and damage, improving oocyte quality | No evidence for the health outcome |
| Higher body weight increases time to pregnancy | Limited Evidence to Suggest AN Association |
| Your lifestyle (work, sleep, stress) is more important than diet when trying to conceive | Insufficient Evidence to Suggest AN Association |
| Eating bananas and sweet potatoes will increase your fertility due to the amount of potassium in the food | No evidence for the health outcome |
| Consuming red meat impacts ovulation and time to pregnancy, and should be limited to 300-500g per week | Insufficient Evidence to Suggest AN Association |
| All women of childbearing age should take a 400-800mcg folic acid supplement daily to reduce the risk of neural tube defects | In Guidelines |
| When trying to conceive, you should limit alcohol to a maximum of 4 standard drinks per week to optimise oocyte health | No evidence for the health outcome |
| Gluten and dairy cause inflammation and should be removed from a good fertility diet as they will impact the health of your eggs | No evidence for the health outcome |
| Omega 3 fatty acids improve egg quality | Insufficient Evidence to Suggest AN Association |
| Antioxidants prevent oxidative stress in oocytes and promote good egg quality | No evidence for the health outcome |
| L-arginine can promote blood flow to the ovaries and improve egg quality by delivering quality nutrients | Insufficient Evidence to Suggest AN Association |
| Vitamin B6 improves egg quality and shortens time to pregnancy | No evidence for the health outcome |
| Bone broth decreases leaky gut, which if left uncontrolled, will increase inflammation and decrease fertility | No evidence for the health outcome |
| Collagen is important for cell health and youth, and should be included in a fertility diet to improve oocyte health | No evidence for the health outcome |
| Spinach supports healthy egg formation and ovulation | No Evidence for the health outcome |
| Walnuts increase blood supply to the uterus, which helps to decrease time to pregnancy | No evidence for the health outcome |
| CoQ10 can improve egg quality, reduce your risk of miscarriage, lower chromosomal abnormalities in embryos and keep your eggs younger for longer | No evidence for the health outcome |
| Pineapples are anti-inflammatory and help to support ovulation and promote good egg health | No evidence for the health outcome |
| Coconut oil is a healthy saturated fat which is important for egg health | No evidence for the health outcome |
| Full fat dairy contains a good amount of saturated fat which plays a key role in boosting fertility. | Limited Evidence to Suggest NO Association |
| Pomegranate is a powerhouse of antioxidants; it helps reduce inflammation in the body and is loaded with other fertility nutrients to improve egg health | No evidence for the health outcome |
| Blueberries contain fibre, which boosts your chances of conceiving by decreasing the amount of circulating oestrogen in your body | No evidence for the health outcome |
| The phytonutrient anthocyanin plays a role in fertility by reducing the chronic inflammation that can play a role in infertility, and hormonal imbalance. | No evidence for the health outcome |
| Probiotics improve your fertility and chances of conception. Fermented foods should be included in a fertility diet. | No evidence for the health outcome |
| You should include 1 tablespoon of apple cider vinegar every morning before food when trying to conceive to balance hormones and improve chances of implantation | No evidence for the health outcome |
| Not eating breakfast leads to inflammation, oxidative stress, and poor egg quality | No evidence for the health outcome |
| Your breakfast should include 25g of protein for optimal fertility. | No evidence for the health outcome |
| Organic foods are better for fertility than commercially farmed foods | No evidence for the health outcome |
| You should avoid dairy, alcohol, processed snacks and refined sugars in the lead up to a pregnancy as they are inflammatory and decrease oocyte health | No evidence for the health outcome |
| Salmon, avocado, red meat, leafy greens and blueberries support fertility and improve egg and sperm health | In Scoping Review |
| Don't eat fruit alone when trying to conceive. Pairing fruit with a protein and a fat will help promote hormone balance and improve reproductive function in your body | No evidence for the health outcome |
| Vegan diets are bad for fertility and pregnancy due to the lack of nutrients and the potential for nutrient deficiencies | No evidence for the health outcome |
| A diet rich in anti-inflammatory foods boosts increases your chances of conceiving | No evidence for the health outcome |
| Your prenatal supplement should include antioxidants to reduce inflammation | Insufficient Evidence to Suggest AN Association |
| Sweet potato helps with conception due to the high amounts of Vitamin A which improve implantation rates | No evidence for the health outcome |
| Adding cinnamon into your meals will reduce inflammation and improve fertility outcomes | No Evidence for the health outcome |
| Take folate as opposed to folic acid as it is better absorbed by your body | No evidence for the health outcome |
| Vitamin C and Vitamin D improve immune function which impacts fertility. Poor immunity negatively affects egg health and pregnancy outcomes | No evidence for the health outcome |
| On a full moon, use filtered water and place jugs in the moonlight. Drinking moon water around ovulation will improve your chances of conceiving that cycle. | No Evidence for the health outcome |
| Eating French fries after ovulation and of an IVF transfer improves chances of implantation. | No Evidence for the health outcome |
| Maca Powder improves thyroid health and contains vitamin C and D which makes it good for egg health and conception | No Evidence for the health outcome |
| Bone broth will decrease time to pregnancy | No evidence for the health outcome |
| Consume organ meat (beef liver especially) 3-4x per week prior to a pregnancy to boost nutrient stores and ensure a healthy pregnancy | No evidence for the health outcome |
| 5 methyl tetrahydrofolate is the best form of folic acid to take when TTC | Insufficient Evidence to Suggest AN Association |
| Massaging pressure points on your wrist and foot can help you fall pregnant faster due to the stress relieving properties of pressure points | No Evidence for the health outcome |
| You shouldn't take a fertility multivitamin supplement when trying to conceive. You should take individual nutrients as they are better absorbed | No evidence for the health outcome |
| FSH (the hormone that triggers your follicles to grow) is 20% higher when you have good, uninterrupted sleep. | Insufficient Evidence to Suggest AN Association |
| Only eat organic fruits and vegetables when trying to conceive as they contain more nutrients than conventionally farmed foods | No Evidence for the health outcome |
| Eating a low protein diet causes infertility | No evidence for the health outcome |
| Stop consuming caffeine. It puts stress on the adrenal glands which causes infertility. | No Evidence for the health outcome |
| Inositol promotes ovulation and improves your chances of conceiving | Insufficient Evidence to Suggest AN Association |
| CoQ10 helps boost egg quality | No evidence for the health outcome |
| A high sugar diet is detrimental to fertility | Insufficient Evidence to Suggest AN Association |
| Make a tea of 1tbsp cloves + 1 cup of water. Drink on an empty stomach first thing in the morning to boost fertility by decreasing inflammation | No Evidence for the health outcome |
| If you are overweight, any type of diet that will allow you to lose weight will improve your fertility | No Evidence for the health outcome |
| Omega 3 fatty acids are rich in antioxidants and are good for fertility | Limited Evidence to Suggest AN Association |
| Sleep can help manage DNA damage to improve oocyte health for a healthy pregnancy and healthy child | No Evidence for the health outcome |
| The best BMI for falling pregnant is 20-25 | In Guidelines |
| Red meat is a type 1 carcinogen and can impact the health of your child long term. Red meat also leads to poor embryo development. | No evidence for the health outcome |
| Don't eat too much fish as fish have environmental toxins from ocean pollution that will negatively affect fertility | No Evidence for the health outcome |
| You need to sleep 7-8 hours per night for normal cell division to happen and reduce inflammation in the body | No evidence for the health outcome |
| Processed foods, animal proteins and refined carbohydrates are inflammatory to the body and increase your chances of infertility | Insufficient Evidence to Suggest AN Association |
| Antioxidant supplements can improve egg quality | No evidence for the health outcome |
| Green leafy vegetables are rich in iron, folic acid and antioxidants and are the best foods when it comes to fertility | Insufficient Evidence to Suggest AN Association |
| Pineapple helps to boost the reproductive hormones in your body thus can help you to conceive, especially if eaten shortly after ovulation | No evidence for the health outcome |
| Salmon is rich in omega 3 fatty acids that helps to regulate oestrogen imbalance as well as increase blood flow to your reproductive organs, promoting good oocyte health | Insufficient Evidence to Suggest AN Association |
| Soy has not been associated with negative reproductive outcomes and can be consumed when trying to conceive | Limited Evidence to Suggest AN Association |
| Eat green vegetables instead of folic acid supplements as they are better absorbed by the body | No evidence for the health outcome |
| Both mother and father should take a folic acid supplement before trying to conceive to reduce risk of congenital abnormalities in the baby | In Guidelines |
| Dairy has vitamin D which is good for fertility and improves the health of oocytes | No Evidence for the health outcome |
| Replace white grains with brown/wholemeal versions to shorten time to pregnancy | Insufficient Evidence to Suggest AN Association |
| Replace white sugar with brown sugar when trying to conceive as the impact on your insulin is less so your oocyte health will improve | No Evidence for the health outcome |
| Low fat dairy is associated with poor reproductive outcomes | Insufficient Evidence to Suggest AN Association |
| Oats increase chances of ovulation and improve implantation | No evidence for the health outcome |
| Avocado contains potassium which helps to regulate blood pressure and therefore improve blood flow to ovaries, enhancing ovulation and egg health | No evidence for the health outcome |
| Consuming folate rather than taking a folic acid supplement is best when trying to conceive as folic acid is synthetic and won't be absorbed by your body | No evidence for the health outcome |
| Low iron levels can be a cause of unexplained infertility | In Guidelines |
| Caffeine intake over 200mg can increase risk of miscarriage | Limited Evidence to Suggest AN Association |
| Folic acid prevents neural tube defects | In Guidelines |
| If you cannot take a folic acid supplement, you can get enough from your food with plenty of leafy greens, beans, eggs, milk daily | Insufficient Evidence to Suggest AN Association |
| Being underweight or overweight can affect your fertility. Aim for a BMI of 20-25 before trying to conceive | In Guidelines |
| Regular exercise and good quality sleep will help reduce stress, which will promote regular ovulation and optimal egg health. | Insufficient Evidence to Suggest AN Association |
| Vitamin D (400-800IU) will help boost egg quality | Insufficient Evidence to Suggest AN Association |
| Spearmint tea improves androgen levels (lowers them) to promote ovulation and decrease time to pregnancy | No evidence for the health outcome |
| Adding matcha green tea to smoothies will boost the antioxidant qualities and help improve egg health | No evidence for the health outcome |
| If you are struggling to conceive, gut health could be to blame. Try the low FODMAP diet to improve gut health and improve fertility | No evidence for the health outcome |
| Red raspberry leaf tea decreases time to pregnancy and improves ovulation and implantation | No evidence for the health outcome |
| Eating fast food increases your time to pregnancy, regardless of BMI | No evidence for the health outcome |
| Acupuncture improves blood flow in the body, including to your ovaries and uterus. This will increase nutrients delivered to your ovaries, improving egg health. | No evidence for the health outcome |
| Intermittent fasting can reduce oxidative damage and inflammation in the body and boost egg quality | No evidence for the health outcome |
| Choose slow carbs - do not go keto when trying to conceive as carbohydrates are important for egg health and implantation | No Evidence for the health outcome |
| Consuming 2 or more servings of seafood per week will help you fall pregnant faster | Limited Evidence to Suggest AN Association |
| Consume full fat dairy to increase chances of ovulation and a successful pregnancy | No evidence for the health outcome |
| You should take a prenatal multivitamin alongside omega 3 and CoQ10 when trying to conceive for the health of your future child | No evidence for the health outcome |
| Omega 3 can optimise egg health and implantation | No Evidence for the health outcome |
| A diet high in trans fats will cause infertility | No evidence for the health outcome |
| Choline is critical for brain and spinal cord development and can reduce neural tube defects | Limited Evidence to Suggest NO Association |
| Lack of choline can affect memory and processing speed of children | Limited Evidence to Suggest NO Association |
| Consuming 1 cup of bone broth daily when trying to conceive over the age of 35 will heal the gut lining and help absorb more nutrients that are critical for pregnancy | No Evidence for the health outcome |
| Low fat dairy can lead to anovulation and increase your time to a successful pregnancy | Limited Evidence to Suggest AN Association |
| 1/4 of your plate at every meal should be wholegrains to increase the chance of a live birth | Limited Evidence to Suggest AN Association |
| Processed soy is inflammatory and decreases fertility | Insufficient Evidence to Suggest AN Association |
| Vitamin D from the sunlight is better than vitamin D from a supplement for egg quality | No evidence for the health outcome |
| The Mediterranean diet will increase your chance of falling pregnant, especially during IVF | In Scoping Review |
| Cold water seafood increases rates of fertility and should be consumed when TTC | No evidence for the health outcome |
| Eating something is better than nothing at breakfast. Not eating breakfast decreases fertility and increases inflammation | No evidence for the health outcome |
| Wholegrain carbohydrates will lead to higher live birth rates. They have antioxidants which are anti-inflammatory and good for preconception diets | Insufficient Evidence to Suggest AN Association |
| Your oocyte quality is best between the ages of 15-21 years | No evidence for the health outcome |
| Yoga will keep your hormones in balance to promote good fertility | No evidence for the health outcome |
| CoQ10 can help boost ovarian reserve and can boost egg quality | No evidence for the health outcome |
| Higher meat intake leads to lower rates of ovulation and pregnancy. | Insufficient Evidence to Suggest AN Association |
| Antioxidants reduce oxidative stress and will protect egg quality | No Evidence for the health outcome |
